# Co-delivery of endoplasmic reticulum-targeted photosensitizer and Ido-1 inhibitor potentiates immunotherapeutic efficacy in triple-negative breast cancer

**DOI:** 10.1016/j.mtbio.2026.103268

**Published:** 2026-05-22

**Authors:** Jia Wang, Fengling Wang, Qiang Zhou, Yue Huang, Mengjun Yu, Shiwen Chen, Jing Gong, Min Yang, Qiang He, Jingbin Huang, Yu Zhao

**Affiliations:** a Department of Pharmacy, University-Town Hospital of Chongqing Medical University, Chongqing, 401331, China; b Department of Pharmacy, The Second Affiliated Hospital of Army Medical University, Chongqing, 400037, China; c Department of Pharmacy, Bishan Hospital of Chongqing Medical University, Chongqing, 402760, China

**Keywords:** Endoplasmic reticulum-targeting, Immunogenic cell death, Indoleamine 2,3-dioxygenase 1 inhibitor, Tumor microenvironment remodeling, Triple-negative breast cancer

## Abstract

Triple-negative breast cancer (TNBC) generally has a poor response to immunotherapy due to low immunogenicity and an immunosuppressive tumor microenvironment. To address this, a multifunctional nanoparticle, TPI@RPB, was constructed with RGD-PEG-BSA as the carrier for tumor-targeted delivery, while co-loading the self-synthesized endoplasmic reticulum (ER) target photosensitizer TSE-Ppa and Indoleamine 2,3-dioxygenase 1 (IDO-1) inhibitor 1-MT. ER-localized reactive oxygen species, generated by TSE-Ppa upon light activation, induce strong immunogenic cell death (ICD). This mechanism facilitates dendritic cell maturation and stimulates the proliferation of CD4^+^/CD8^+^ T cells, thereby boosting immunogenicity. Meanwhile, IDO-1 inhibition suppresses tryptophan metabolism-driven immune evasion, thereby reshaping the tumor microenvironment to favor antitumor immunity. *In vivo*, TPI@RPB not only demonstrates active tumor targeting but also significant antitumor efficacy with a favorable safety profile; specifically, tumor weight reduced to 13.70%, intratumoral CD8^+^ T-cell infiltration increased 1.92-fold, Treg cells decreased to 51.7%, and splenic effector-memory T cells increased 4.70-fold of control, with no observable systemic toxicity. These results suggest that ER-targeted PDT combined with IDO-1 blockade effectively remodels the tumor microenvironment and elicits durable antitumor immunity in TNBC.

## 1 Introduction

Triple-negative breast cancer (TNBC) is a highly invasive and metastatic breast cancer subtype with the characteristics of a lack of estrogen receptor, progesterone receptor, and human epidermal growth factor receptor 2 [1]. Because effective targeted treatments are lacking, the current treatment mainly relies on surgery combined with chemotherapy and radiotherapy [2,3]. However, the clinical effect is often not ideal, manifested as a high recurrence rate, limited treatment effect, and poor overall survival rate [4,5], which highlights the urgent need to develop new treatment strategies.

Immunotherapy has expanded the therapeutic landscape of TNBC by activating the host immune system to fight tumor cells [6,7]. Nevertheless, its clinical benefit is often restricted by the intrinsically low immunogenicity of TNBC and a strongly immunosuppressive tumor microenvironment [8,9]. As a cutting-edge technology that can induce immunogenic cell death (ICD), photodynamic therapy (PDT) has received wide attention in recent years and is considered a promising auxiliary treatment for TNBC [10,11]. During PDT, photosensitizers are activated by light irradiation at a specific wavelength to generate reactive oxygen species (ROS). When ROS production occurs in endoplasmic reticulum (ER) -associated regions, the resulting oxidative stress can amplify ER stress and promote ICD [12]. ICD is accompanied by the exposure or release of damage-associated molecular patterns (DAMPs), including calreticulin (CRT), high-mobility group box 1 (HMGB1), and adenosine triphosphate (ATP). By facilitating antigen presentation in dendritic cells (DCs) and augmenting cytotoxic T lymphocytes (CTLs) proliferation, these signals activate the host immune response [[13], [14], [15]].

However, the therapeutic efficacy of PDT is not determined solely by the total amount of ROS generated, but is also closely associated with the intracellular sites of ROS action and their local intensity [16]. Due to the short lifetime and limited diffusion of ROS, diffusely distributed photosensitizers may dilute oxidative stress, preventing it from reaching the local threshold needed to overcome immune tolerance and thereby limiting the immune activation of conventional PDT [17]. The ER, a central organelle responsible for protein folding, calcium homeostasis, and oxidative stress sensing, serves as a critical link between PDT-induced oxidative damage and ICD initiation. Previous studies have shown that ER stress functions as an important upstream event in ICD, promoting DAMP-associated responses such as CRT exposure, ATP release, and HMGB1 release [[18], [19], [20], [21], [22]]. Therefore, precise delivery of photosensitizers to ER-associated regions, where localized high-intensity ROS can be generated upon irradiation, may enhance PDT-induced ICD at the subcellular level [23]. N-tosylethylenediamine (TSE) has been reported to interact with ATP-sensitive potassium channels located on the ER membrane, thereby conferring ER-targeting capability to conjugated molecules [24]. When coupled with the classic photosensitive agent Pyropheophorbide-a (Ppa), the resulting TSE-Ppa demonstrates enhanced ER accumulation and stress induction, leading to more potent PDT-mediated cell death and increased tumor immunogenicity.

Despite these advances, PDT alone is insufficient to overcome the immunosuppressive milieu of TNBC. ICD triggered by PDT can stimulate the release of Interferon-γ (IFN-γ) by CTLs; however, IFN-γ indirectly enhances multiple immune checkpoint pathways at the same time, including the key node of upregulation of Indoleamine 2,3-dioxygenase 1 (IDO-1) expression [25]. IDO-1 catalyzes the conversion of tryptophan (Trp) into kynurenine (Kyn): Trp depletion impairs CD8^+^ T-cell effector function, whereas Kyn accumulation promotes regulatory T cells (Tregs) activation, and finally inhibits CTL-mediated anti-tumor immunity [26,27]. Thus, metabolic immune escape mediated by IDO-1 represents a critical barrier that limits the durability of PDT-elicited immune responses. The competitive IDO-1 inhibitor 1-methyl-L-tryptophan (1-MT) can occupy the catalytic site of IDO-1, block Trp catabolism, and partially reverse tumor-driven immunosuppression, thereby offering a rational means to sustain and amplify ICD-triggered antitumor immunity [28].

Nanodelivery systems provide a practical basis for integrating these therapeutic strategies. Nanocarriers can improve the dispersibility and stability of hydrophobic agents while promoting their accumulation at tumor sites [29,30]. In addition, RGD modification can enhance the recognition and internalization of the carrier by tumor cells and receptor-expressing tumor vasculature [31]. Accordingly, co-delivering an ER-targeted photosensitizer and an IDO-1 inhibitor through a single nanoplatform may couple enhanced tumor delivery with ER-localized stress amplification and immunosuppression relief.

Herein, we constructed a multifunctional nanoplatform, TPI@RPB, by co-loading TSE-Ppa and 1-MT into an RGD-PEG-BSA carrier. In this system, TSE-Ppa serves as an ER-targeted photosensitizer to concentrate ROS generation in ER-associated regions and amplify ICD, while 1-MT is incorporated to inhibit the IDO-1 pathway and remodel the immunosuppressive tumor microenvironment. The RGD-PEG-BSA carrier further facilitates tumor-associated delivery through RGD-mediated recognition of integrin-expressing tumor cells and tumor vasculature. Thus, TPI@RPB integrates tumor-targeted delivery at the tissue level, ER-focused oxidative stress amplification at the subcellular level, and immunometabolic modulation within a single therapeutic platform. By addressing both insufficient immune activation in conventional PDT and tumor-associated immunosuppression, this strategy provides a rational basis for photodynamic immunotherapy against TNBC.

## 2 Results and discussion

### 2.1 Nanoparticle preparation and characterization

First, we employed a TSE-derived moiety, which selectively binds to ATP-sensitive potassium channels on the ER membrane, as an ER-targeting ligand. By conjugating TSE to the porphyrin-based photosensitizer Ppa, we constructed a novel ER-localizing photosensitizer (TSE-Ppa), aiming to enhance PDT-triggered ICD and downstream immune activation (Fig. S1, Supporting Information) [32]. The successful synthesis of TSE-Ppa was confirmed by ^1^H NMR (Fig. S2, Supporting Information) and further verified by Matrix-Assisted Laser Desorption/Ionization Time-of-Flight Mass Spectrometry (MALDI-TOF MS), which showed a molecular ion peak consistent with the calculated mass of TSE-Ppa (Fig. S3, Supporting Information).

**Fig. 1:**
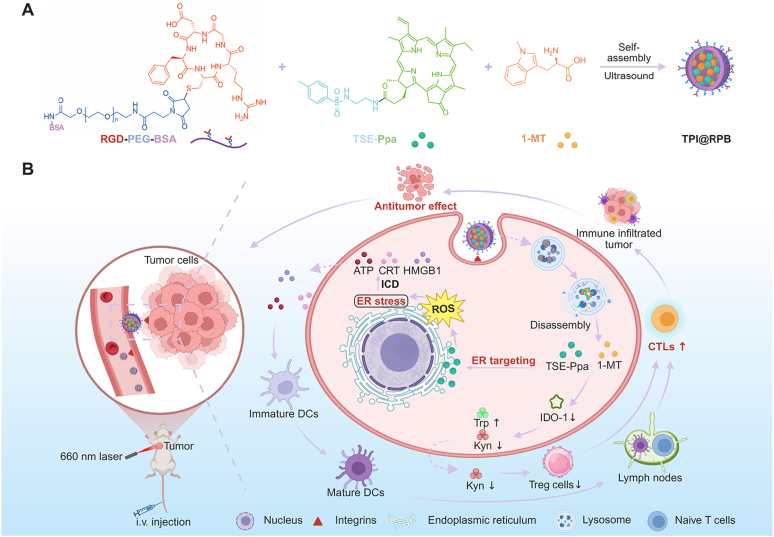
Schematic depiction of TPI@RPB remodeling the tumor-immunosuppressive microenvironment to enhance immunotherapy efficacy in TNBC. (A) Preparation route for TPI@RPB. (B) Illustration of the mechanism of TPI@RPB-mediated combined PDT and immune checkpoint blockade therapy for TNBC upon 660 nm laser irradiation.

Next, the tumor-targeting carrier RGD-PEG-BSA was synthesized through a two-step acid-amine condensation reaction (Fig. S4, Supporting Information) [33]. The successful conjugation of RGD-PEG-BSA was characterized and confirmed by ^1^H NMR, Fourier Transform Infrared Spectroscopy (FTIR), and MALDI-TOF MS. Compared with native BSA, the ^1^H NMR spectrum of RGD–PEG–BSA shows new aromatic signals from the RGDfC phenyl ring and PEG-chain resonances, while the diagnostic maleimide [34] and NHS-ester [35] peaks are largely absent, consistent with successful conjugation (Fig. S5, Supporting Information). FTIR results showed a coherent trend: RGD-PEG-BSA largely retained the characteristic bands of BSA—particularly amide I/II (∼1650/1540 cm^−1^) [36,37], indicating preservation of the protein secondary structure—and, on this basis, exhibited pronounced PEG signatures (∼1100 cm^−1^, C–O–C; ∼2880 cm^−1^, C–H) [38]. Because the amide vibrations of the RGD peptide overlap extensively with the BSA amide bands, the introduction of RGD is supported indirectly by the loss of the maleimide = C–H out-of-plane vibration (∼690 cm^−1^ [39]), consistent with thiol–maleimide coupling (Fig. S6, Supporting Information). MALDI–TOF MS was performed to verify the RGD-PEG intermediate and RGD-PEG-BSA conjugation. The RGD-PEG intermediate showed a characteristic PEG-distributed peak centered at *m*/*z* 5789.2149, indicating successful formation of the RGD-modified PEG intermediate. Compared with native BSA, which exhibited a main peak at approximately 66.6 kDa, RGD-PEG-BSA displayed a new peak at approximately 72.2 kDa, with a mass increase of about 5.6 kDa. This shift is consistent with the conjugation of approximately one RGD-PEG5000 chain to each BSA molecule. Based on the initial RGD-PEG/BSA feeding ratio of 5:1, the average grafting number corresponded to an estimated RGD-PEG utilization of approximately 20%. The MALDI-TOF MS results support the formation of RGD-PEG-BSA conjugates, although residual unmodified BSA was still present in the final preparation (Fig. S7, Supporting Information) (see Fig. 1).

**Fig. 2:**
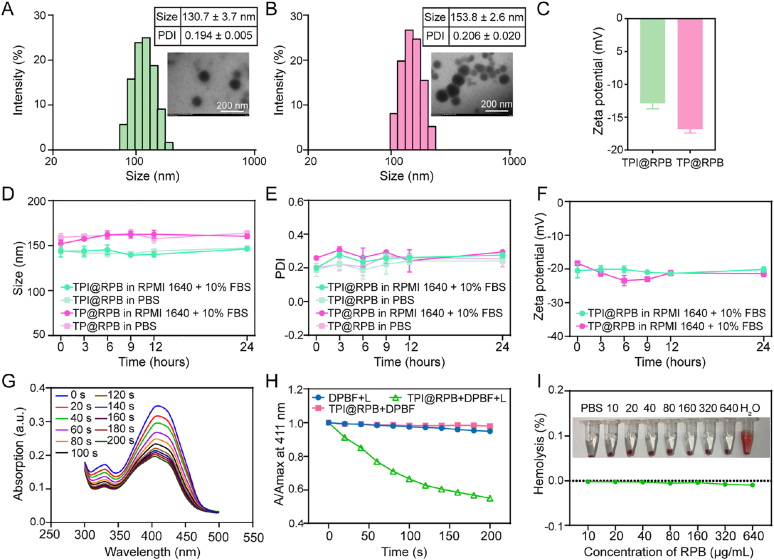
Characterization of TPI@RPB. Hydrodynamic diameter and representative TEM images of TPI@RPB (A) and TP@RPB (B), scale bar = 200 nm. (C) Zeta-potential of TPI@RPB and TP@RPB. Changes in hydrodynamic diameter (D), PDI (E) of TPI@RPB and TP@RPB in PBS and RPMI 1640 medium with 10% serum (37 °C, 120 rpm) over 24 h. (F) Zeta-potential of TPI@RPB and TP@RPB in RPMI 1640 medium with 10% serum (37 °C, 120 rpm) over 24 h. (G) Time-dependent changes in DPBF quenching at 300–500 nm under laser irradiation (660 nm, 0.5 W/cm^2^) in the TPI@RPB + L group. (H) Degradation rate of DPBF under the action of ROS in each group. (I) The hemolysis rate of RGD-PEG-BSA at different concentrations was used to evaluate hemocompatibility.

TPI@RPB and TP@RPB were then produced by ultrasonic self-assembly. As shown in Fig. 2A–C, TPI@RPB exhibited a spherical morphology with an average hydrodynamic diameter of 130.7 ± 3.7 nm, a PDI of 0.194 ± 0.005, and a zeta potential of −12.9 ± 0.8 mV. In contrast, TP@RPB was slightly larger (153.8 ± 2.6 nm, PDI: 0.206 ± 0.020) and had a more negative surface charge (−16.8 ± 0.6 mV), suggesting that 1-MT helped stabilize the nanoparticles. Similar results were obtained in transmission electron microscopy (TEM), showing that both nanoparticles exhibited a uniform spherical morphology, and that TPI@RPB exhibited less aggregation than TP@RPB. TPI@RPB exhibited high encapsulation efficiency (EE%) relative to TSE-Ppa (90.05 ± 5.14%) and 1-MT (85.35 ± 0.95%), with loading efficiency (LE%) of 13.32 ± 0.65% and 12.63 ± 0.10%, respectively. TP@RPB showed comparable results (LE%: 15.76 ± 0.08%, EE%: 93.56 ± 0.57%) (Fig. S8, Supporting Information). The stability of TPI@RPB and TP@RPB was then investigated when stored at room temperature for 7 days. Both formulations were stable at room temperature over 7 days, with only minor changes in particle size, PDI, and Zeta potential (Fig. S9, Supporting Information). After confirming the storage stability of TPI@RPB and TP@RPB, their short-term colloidal stability under cell-culture-relevant conditions was further evaluated. As shown in Fig. 2D–F, both TPI@RPB and TP@RPB showed negligible changes in particle size and PDI in these media, indicating the absence of obvious aggregation during incubation. In serum-containing medium, the apparent zeta potentials of both formulations shifted to approximately −20 mV and remained stable, which may be attributed to serum protein adsorption and ionic shielding. These results demonstrate that TPI@RPB and TP@RPB maintain favorable short-term colloidal stability under cell culture conditions, supporting their use in subsequent *in vitro* experiments.

To investigate the effect of TPI@RPB on ROS generation, the UV absorption spectra of the TPI@RPB and DPBF co-incubated groups showed a significant decreasing trend within 2 min after laser irradiation (Fig. 2G and H), whereas the UV absorption spectra of the DPBF + L and DPBF + TPI@RPB groups showed no significant change (Fig. S10, Supporting Information), indicating that TPI@RPB was more likely to produce ROS upon laser irradiation.

To assess the hemocompatibility of RGD-PEG-BSA, mouse erythrocytes were exposed to increasing concentrations (10–640 μg/mL), which resulted in a concentration-dependent hemolysis of less than 5% at all doses tested (Fig. 2I). These results indicated that TPI@RPB had good hemocompatibility and confirmed its suitability for *in vivo* use.

The release behavior of 1-MT from TPI@RPB was evaluated under different pH and irradiation conditions. As shown in Figure S11, 1-MT release from TPI@RPB exhibited a pH-dependent profile. The cumulative release reached approximately 30% at pH 7.4, but increased to about 50% at pH 6.5 and 55% at pH 5.0 after 48 h. In contrast, laser irradiation produced no obvious additional release compared with the corresponding non-irradiated groups. These results indicate that TPI@RPB can retain 1-MT under physiological conditions while promoting its release in acidic tumor-related environments, supporting subsequent IDO-1 inhibition during PDT-based immunotherapy. The accurate quantification of TSE-Ppa was compromised under acidic conditions, which hindered reliable determination of its release behavior [40].

### 2.2 Targeted cellular delivery and ER-associated photodynamic activation

**Fig. 3:**
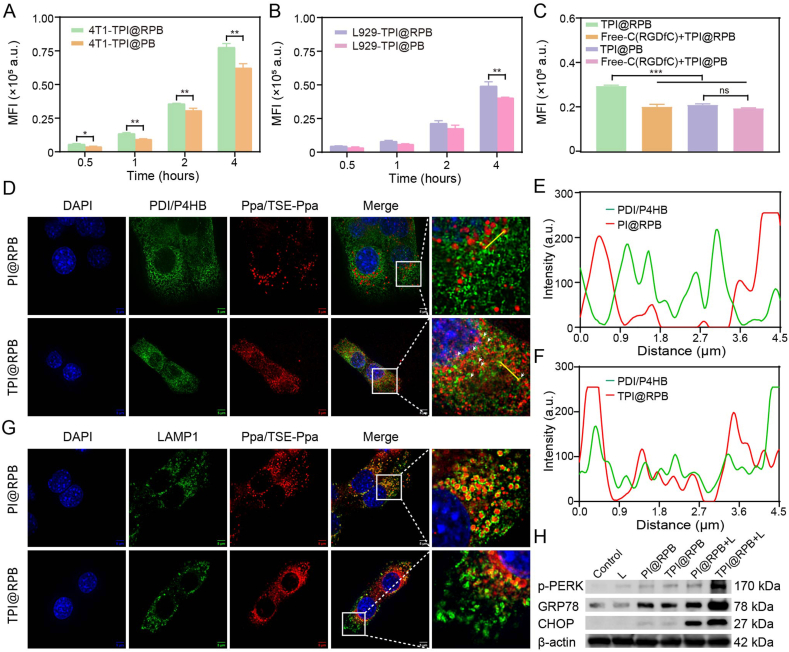
RGD-mediated cellular uptake, ER-associated localization, and light-triggered ER stress of TPI@RPB in 4T1 cells. (A) Quantification of intracellular uptake of TPI@RPB and TPI@PB in 4T1 cells by FCM at various time points (N = 3). (B) Quantification of intracellular uptake of TPI@RPB and TPI@PB in L929 cells by FCM at various time points (N = 3). (C) Competitive uptake of TPI@RPB and TPI@PB in 4T1 cells with or without free C (RGDfC) pretreatment, quantified by FCM after 2 h incubation (N = 3). (D) Super-resolution confocal imaging of ER-targeting by TPI@RPB and PI@RPB in 4T1 cells after 4 h incubation. PDI/P4HB was used for ER labeling, scale bar = 5 μm. Fluorescence intensity profiles are shown along the lines in Fig. 3D. The red and green curves correspond to the fluorescence signals of PI@RPB (E) and TPI@RPB (F) and the ER, respectively. (G) Super-resolution confocal imaging of the lysosomal distribution of TPI@RPB and PI@RPB in 4T1 cells after 4 h incubation. LAMP1 was used for lysosomal labeling, scale bar = 5 μm. (H) ER stress-related protein expression in 4T1 cells after treatment with each group, analyzed by western blot. Data were shown as mean ± SD, ns means no statistical significance, ∗ is p < 0.05, ∗∗ is p < 0.01, ∗∗∗ is p < 0.001. (For interpretation of the references to colour in this figure legend, the reader is referred to the Web version of this article.)

The cellular uptake of TPI@RPB was first evaluated in 4T1 cells by flow cytometry (FCM). As shown in Fig. 3A, both TPI@RPB and the non-RGD-modified TPI@PB exhibited time-dependent internalization within 0.5–4 h. TPI@RPB showed significantly higher intracellular fluorescence than TPI@PB at each tested time point, indicating that RGD modification facilitated nanoparticle uptake in 4T1 cells. Confocal laser scanning microscopy (CLSM) images further showed time-dependent intracellular accumulation of both formulations at 1 and 2 h. Quantitative analysis revealed no significant difference between TPI@RPB and TPI@PB at 1 h, but a significantly higher fluorescence intensity of TPI@RPB at 2 h (Fig. S12). This trend was consistent with the FCM results, although the difference was less pronounced in CLSM quantification due to the limited number of imaging fields. Thus, FCM was used as the primary quantitative evidence for assessing RGD-mediated uptake enhancement, whereas CLSM served as supportive imaging evidence.

To further evaluate whether RGD modification preferentially enhanced nanoparticle uptake in tumor cells, L929 fibroblasts were selected as a normal-cell control for comparison. In L929 cells, the uptake of both formulations also increased over time, but the overall fluorescence intensity was lower than that observed in 4T1 cells (Fig. 3B), suggesting a relatively preferential uptake profile of TPI@RPB in tumor cells. To further verify the role of RGD-mediated recognition, free C (RGDfC) was used for competitive inhibition. Pretreatment with free C (RGDfC) markedly reduced the uptake of TPI@RPB in 4T1 cells, whereas no significant effect was observed for TPI@PB (Fig. 3C), supporting that the enhanced uptake of TPI@RPB was at least partially mediated by RGD-related receptor recognition. Additional uptake inhibition assays showed that TPI@RPB internalization was largely energy-dependent and involved clathrin-mediated and caveolae/lipid raft-associated endocytosis, providing a cellular entry basis for subsequent intracellular trafficking (Fig. S13).

After cellular uptake was confirmed, the intracellular localization of TPI@RPB was examined. Protein disulfide isomerase/P4HB (PDI/P4HB) immunofluorescence staining was used to label the ER. Compared with PI@RPB, TPI@RPB displayed a more evident distribution along or adjacent to the PDI/P4HB-positive ER network after 4 h incubation (Fig. 3D). This was further supported by fluorescence line profile analysis, which showed a closer spatial association between TPI@RPB fluorescence and the PDI/P4HB signal compared with PI@RPB (Fig. 3*E*–F). Consistently, thresholded Manders analysis showed markedly higher colocalization coefficients for TPI@RPB than for PI@RPB, with tM1/tM2 increasing from 0.003/0.330 for PI@RPB to 0.628/0.669 for TPI@RPB. These results support the enhanced ER-associated localization of TPI@RPB. This behavior can be attributed to the TSE moiety in TSE-Ppa, which confers ER-targeting capability. LAMP1 immunofluorescence staining was further performed to examine lysosomal distribution. PI@RPB showed more apparent overlap with LAMP1-positive puncta, whereas TPI@RPB exhibited a broader intracellular distribution with less obvious lysosomal confinement after 4 h incubation (Fig. 3G). These results suggest that TPI@RPB was not predominantly retained in lysosomes and could achieve increased ER-associated distribution after internalization.

Finally, western blotting was used to determine whether ER-associated localization could be translated into functional ER stress. Without irradiation, PI@RPB and TPI@RPB induced only limited changes in ER stress-related proteins. After 660 nm laser irradiation, PI@RPB + L increased p-PERK, GRP78, and CHOP expression to some extent, whereas TPI@RPB + L produced the most pronounced upregulation (Fig. 3H). These results demonstrate that RGD modification enhanced cellular uptake of TPI@RPB, while TSE-Ppa promoted ER-associated localization and amplified light-triggered ER stress, establishing the cellular basis for subsequent ER-directed photodynamic immunotherapy.

### 2.3 Tumor cell killing and ICD-mediated immune activation in vitro

Having validated the ER-targeting function of TSE-Ppa in the preceding section, we next used TP@RPB as the TSE-Ppa-based PDT reference formulation and TPI@RPB as the combined formulation to assess whether 1-MT incorporation could further support PDT-mediated antitumor and immune effects.

**Fig. 4:**
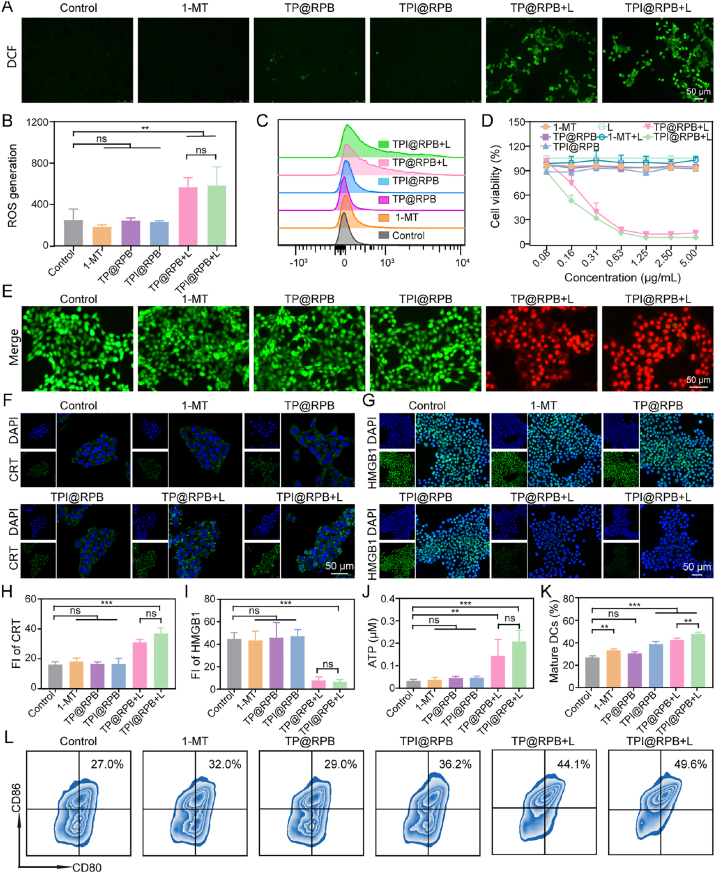
ER-targeted photodynamic ROS generation by TPI@RPB drives tumor cell killing and antitumor immune activation *in vitro*. (A) Visualization of intracellular ROS levels in 4T1 cells using IFM. Green fluorescence indicates DCF formation due to ROS-mediated oxidation of DCFH-DA, scale bar = 50 μm. (B–C) Intracellular ROS generation in each treatment group using FCM (N = 3). (D) 4T1 cell viability in each treatment group was determined using the CCK-8 assay (N = 3). (E) 4T1 cells under various treatments were evaluated using a live/dead assay, scale bar = 50 μm. (F) CLSM images of surface CRT exposure in 4T1 cells after treatment with each group, scale bar = 50 μm. (G) CLSM images of HMGB1 release in 4T1 cells upon treatment with each treatment group, scale bar = 50 μm. (H) Statistical analysis of CRT mean fluorescence intensity (N = 3). (I) Statistical analysis of HMGB1 mean fluorescence intensity (N = 3). (J) Extracellular ATP level in each treatment group (N = 3). (K) FCM analysis of DC maturation induced by ICD in each treatment group (N = 3). (L) Frequency of mature DCs, identified within the CD11c^+^ cell population by a gating strategy. Data were shown as mean ± SD, ns means no statistical significance, ∗∗ is *p* < 0.01, ∗∗∗ is *p* < 0.001. (For interpretation of the references to colour in this figure legend, the reader is referred to the Web version of this article.)

Intracellular ROS production was detected using the ROS-responsive probe DCFH-DA. Inverted fluorescence microscope (IFM) analysis showed a strong green fluorescent signal corresponding to DCFH in the TPI@RPB + L treatment group (Fig. 4A). FCM analysis also showed that the intracellular fluorescence intensity of the TPI@RPB + L group was approximately 2.5-fold higher than the TPI@RPB group (Fig. 4B and C). These data suggest that light irradiation with TPI@RPB effectively induced ROS formation and contributed to the subsequent cytotoxic effect on 4T1 cells.

Before the *in vivo* experiments, the cytotoxicity of TPI@RPB with CCK-8 was examined in 4T1 tumor cells. The free 1-MT, TP@RPB, and TPI@RPB groups showed no significant cytotoxic effect on 4T1 cells at concentrations ranging from 0.08 to 5.00 μg/mL (Fig. 4D). Notably, the TP@RPB + L and TPI@RPB + L groups showed a concentration-dependent decrease in cell viability. In detail, at the same TSE-Ppa concentration of 1.25 μg/mL, laser irradiation alone resulted in a cell viability of 105.3 ± 3.4%, while treatment with 1-MT alone and 1-MT + L groups led to viabilities of 93.3 ± 3.3% and 100.0 ± 7.2%, respectively. Similarly, 87.6 ± 1.2% of 4T1 cells survived when incubated with TPI@RPB. In contrast, cell survival in the TPI@RPB + L group was significantly reduced to 8.1 ± 0.4% under the same conditions, indicating a therapeutic effect of TSE-Ppa-mediated PDT. Analysis of live/dead cell staining also showed relevant results. In all non-irradiated groups, the level of green fluorescence was comparable to control. In contrast, the laser-treated group displayed elevated red fluorescence and concurrently decreased green fluorescence (Fig. 4E).

Photodynamically generated ROS induce tumor cell cytotoxicity and activate ER stress, thereby initiating ICD-associated antitumor immune responses [41]. In order to evaluate whether TPI@RPB can enhance ICD, this study detected ICD-related markers, including CRT, HMGB1, and ATP. As shown in Fig. 4F, TP@RPB + L and TPI@RPB + L markedly increased surface CRT, whereas free 1-MT, TP@RPB, or TPI@RPB alone did not. Quantitative analysis of CRT fluorescence images confirmed consistent results (Fig. 4H). In addition, HMGB1 immunofluorescence showed that TP@RPB + L and TPI@RPB + L significantly promoted extracellular HMGB1 release (Fig. 4G), which was further supported by quantitative fluorescence analysis (Fig. 4I). Consistently, ELISA analysis of the culture supernatants revealed markedly elevated extracellular HMGB1 levels in the TP@RPB + L and TPI@RPB + L groups compared with the control and non-irradiated groups, while no significant difference was observed between the two irradiated nanoparticle groups (Fig. S14). To further validate the ability of TPI@RPB to elicit extensive ICD, the ATP released by tumor cells is quantitatively determined by the ATP detection method. The ATP concentration in the cell culture supernatant of the TPI@RPB + L treatment group reached 0.21 ± 0.05 μM, and the release level was about 4.4 times higher than that of the TPI@RPB group (Fig. 4J). Importantly, there was no statistically significant difference between the TP@RPB + L group and the TPI@RPB + L group in terms of CRT surface exposure, HMGB1 release, and ATP secretion. A possible explanation was that 1-MT, an IDO-1 inhibitor, had no significant effect on ICD induction.

DCs are important mediators of antigen presentation and are essential for T cell priming. Highly immunogenic tumor cells can promote DC maturation and thereby initiate and enhance adaptive immune responses [42,43]. As shown in Fig. 4K and L, bone marrow-derived dendritic cells (BMDCs) co-cultured with 4T1 cells and pre-treated with TPI@RPB + L group showed the highest immunogenicity, as evidenced by an increased percentage of mature DCs (CD11c^+^CD80^+^CD86^+^, 47.73 ± 1.63%). In contrast, only 38.80 ± 2.26% of mature DCs were observed when TPI@RPB-treated 4T1 cells and BMDCs were cultured in the absence of laser irradiation, suggesting that photodynamic activation significantly promotes ICD-mediated DC maturation.

### 2.4 Inhibiting Ido-1 in vitro

**Fig. 5:**
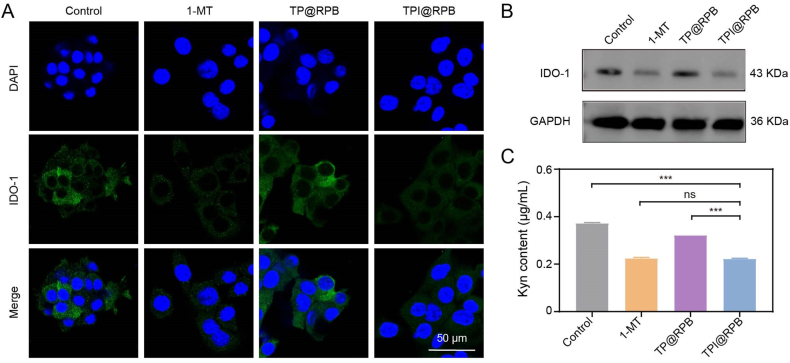
IDO-1 Inhibition of TPI@RPB. (A) Immunofluorescence images of IDO-1 in 4T1 cells treated with each group, scale bar = 50 μm. (B) IDO-1 expression in 4T1 cells treated with each group was examined by Western blot. (C) The concentration of kynurenine in the 4T1 cell culture medium of each treatment group (N = 3). Data were shown as mean ± SD, ns means no statistical significance, ∗∗∗ is p < 0.001.

IDO-1 functions as an important metabolic immune checkpoint by catalyzing the conversion of tryptophan (Trp) into kynurenine (Kyn). Trp depletion impairs CD8^+^ T-cell effector function, whereas Kyn accumulation promotes regulatory T-cell activation, thereby suppressing CTL-mediated antitumor immunity. As a Trp analogue, 1-MT competitively inhibits IDO-1 catalytic activity and attenuates IDO-1-mediated Trp–Kyn metabolic immunosuppression. To establish an IDO-1-high cellular model, 4T1 cells were prestimulated with IFN-γ for 24 h before nanoparticle treatment. Immunofluorescence staining was first performed to examine the IDO-1-associated cellular signal [44]. Compared with the IFN-γ-stimulated control group, 1-MT-containing treatments reduced the IDO-1 fluorescence signal, and TPI@RPB showed an inhibitory profile comparable to that of free 1-MT (Fig. 5A). Consistently, western blot analysis [45] showed a reduction in IDO-1 protein level under the same treatment conditions (Fig. 5B). Because 1-MT primarily acts by inhibiting IDO-1 enzymatic activity rather than directly blocking IDO-1 protein expression, Kyn production was further quantified by HPLC to evaluate functional IDO-1 inhibition [46,47]. As shown in Fig. 5C, 1-MT-containing groups significantly suppressed Kyn generation. In particular, TPI@RPB decreased the Kyn concentration by 30.99% compared with TP@RPB. In parallel, immunofluorescence and western blot analyses showed reduced IDO-1-associated protein signals, while HPLC quantification confirmed decreased Kyn production, supporting functional blockade of the IDO-1 pathway.

### 2.5 Tumor targeting in vivo

**Fig. 6:**
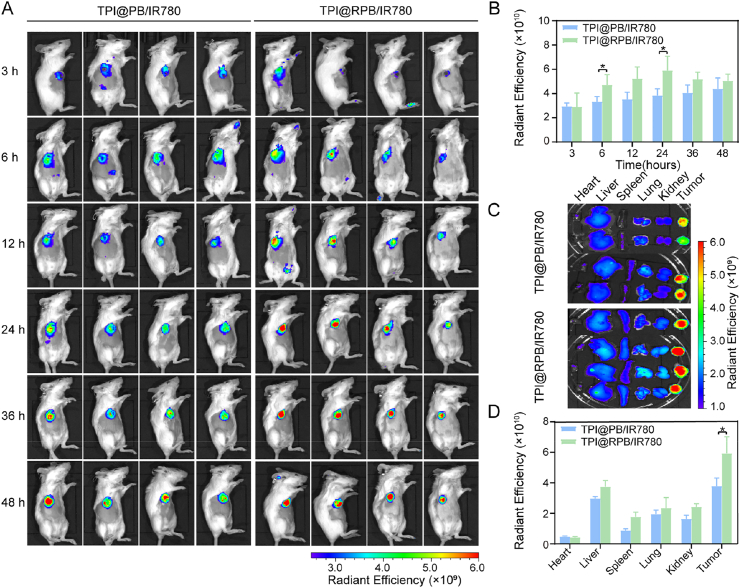
Biodistribution of the TPI@RPB *in vivo*. (A) *In vivo* fluorescence imaging of the biodistribution of TPI@PB/IR780 and TPI@RPB/IR780 in mice with 4T1 tumor (IR780 dose of 1 mg/kg), along with quantitative fluorescence intensity analysis (B) (N = 4). (C) Tumors and major organs were imaged *ex vivo* 48 h after injection, along with quantitative fluorescence intensity analysis (D) (N = 4). Data were shown as mean ± SD, ∗ is *p* < 0.05.

To evaluate the *in vivo* tumor accumulation of RGD-modified nanoparticles, NIR fluorescence imaging was performed in 4T1 tumor-bearing mice after intravenous injection of IR780-loaded TPI@PB or TPI@RPB [48]. As shown in Fig. 6A, fluorescence signals were gradually enriched at the tumor sites in both groups, indicating tumor accumulation of the nanoparticles after systemic administration. Quantitative analysis of the tumor regions further showed that TPI@RPB/IR780 exhibited higher fluorescence intensity than TPI@PB/IR780 during the observation period, with significant differences observed at 6 and 24 h (Fig. 6B). The tumor fluorescence of TPI@RPB/IR780 remained evident up to 48 h, suggesting prolonged tumor retention.

*Ex vivo* imaging of major organs and tumors at 48 h further confirmed the biodistribution profile of both formulations (Fig. 6C). Semi-quantitative analysis showed that the tumor fluorescence intensity of TPI@RPB/IR780 was significantly higher than that of TPI@PB/IR780 (Fig. 6D), supporting the enhanced tumor accumulation of RGD-modified nanoparticles. Meanwhile, relatively strong fluorescence signals were also observed in metabolically active organs such as the liver and kidney. This phenomenon does not contradict the tumor-targeting effect of RGD modification, because active targeting generally improves relative tumor accumulation rather than conferring absolute tumor-exclusive distribution. After systemic administration, nanoparticles are still subject to blood circulation, protein adsorption, mononuclear phagocyte system uptake, hepatic metabolism, and renal-associated clearance. In addition, RGD modification may alter the nanoparticle biointerface, including protein corona formation, vascular interaction, and organ-level retention/clearance, which could contribute to the moderately increased fluorescence observed in non-tumor organs such as the kidney [49]. Therefore, the increased kidney signal should be interpreted as part of the systemic biodistribution and clearance process rather than evidence against tumor targeting.

### 2.6 Antitumor experiment in vivo

**Fig. 7:**
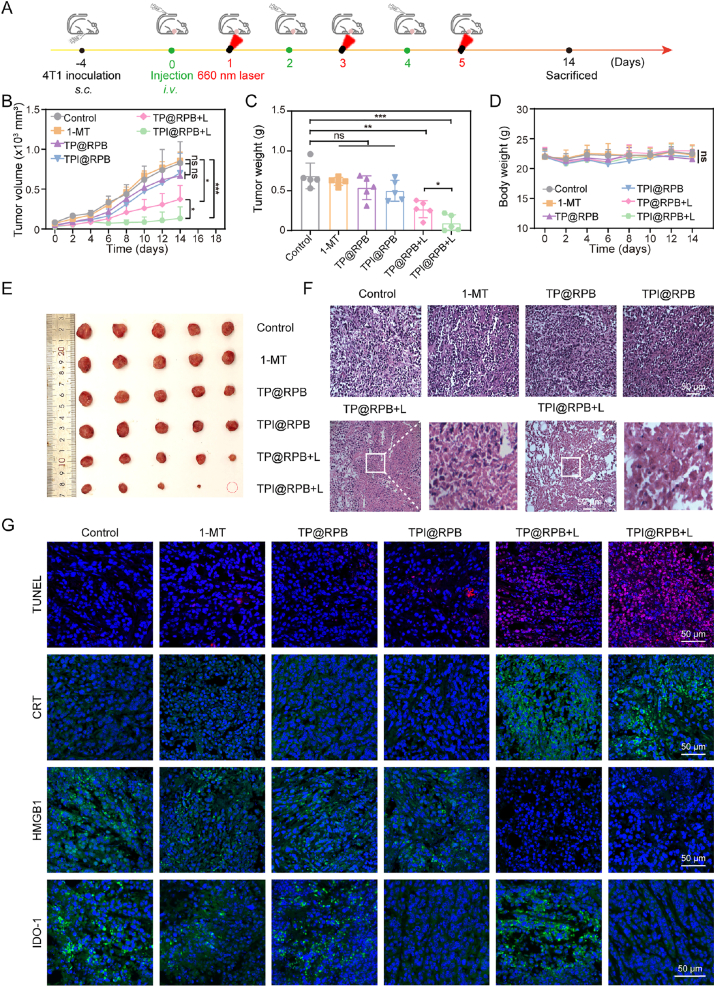
Evaluation of the antitumor efficacy of TPI@RPB. (A) Treatment timeline for TPI@RPB-based combination treatment. (B) Across different treatments, tumor volume in mice (N = 5). (C) The weights of excised tumors in different groups (N = 5). (D) Changes in mouse body weight across different treatments (N = 5). (E) The photos of excised tumors in different groups. (F) H&E of slices of excised tumors in each treatment group, scale bar = 50 μm. (G) TUNEL, CRT, HMGB1, and IDO-1 staining of slices of excised tumors in each treatment group, scale bar = 50 μm. Data were shown as mean ± SD, ns means no statistical significance, ∗ is *p* < 0.05, ∗∗ is *p* < 0.01, ∗∗∗ is *p* < 0.001.

To authenticate the therapeutic benefit of combining photodynamic therapy with IDO-blockade immunotherapy, we established a subcutaneous 4T1 tumor model in BALB/c mice. As shown in Fig. 7A, the drug administration regimen for 4T1 tumor-bearing mice is illustrated. The 1-MT group, TP@RPB, and TPI@RPB did not show significant tumor inhibition relative to the control, likely due to the rapid systemic clearance of the free drug and the inability of non-irradiated nanoparticles to trigger ICD. Strikingly, TPI@RPB + L demonstrated the most potent antitumor effect, with significant reductions in tumor volume (Fig. 7B and Fig. S15) and weight (Fig. 7C), and representative excised tumor images (Fig. 7E). Specifically, on treatment day 14, the TPI@RPB + L group showed tumor volumes at 16.07% of those in controls. A similar trend was observed in tumor weight, which was only 13.70% of the control group. Notably, all treatment groups exhibited no obvious systemic toxicity, as supported by stable body weights (Fig. 7D).

H&E staining showed extensive tumor necrosis and nuclear ablation in the TPI@RPB + L group (Fig. 7F). Furthermore, obvious apoptosis of tumor cells in the TPI@RPB + L group was confirmed by TUNEL staining results (Fig. 7G). The excellent anti-tumor effect of the TPI@RPB + L group confirmed the therapeutic advantages of combining photodynamic therapy and IDO inhibitory immunotherapy.

To clarify the mechanism underlying the enhanced anti-tumor effect observed in the TPI@RPB + L group, we first tested whether this treatment induces ICD in tumor cells *in vivo*. CLSM studies of isolated tumor sections showed that treatment with the TP@RPB + L and TPI@RPB + L groups significantly increased CRT membrane translocation and HMGB1 secretion (Fig. 7G). These results confirm that the TP@RPB + L and TPI@RPB + L groups effectively induced ICD in tumor tissue. In addition, CLSM analysis of tumor sections was performed to assess IDO-1 expression. Compared to the control group, treatment with free 1-MT led to a mild reduction in green fluorescence associated with IDO-1. In contrast, IDO-1 signal intensity was significantly reduced in the TPI@RPB and TPI@RPB + L groups (Fig. 7G). This reduction in IDO-1-associated fluorescence may be related to enhanced intratumoral delivery of 1-MT by nanoparticle encapsulation and should be interpreted together with the Kyn assay as evidence of IDO-1 pathway inhibition.

**Fig. 8:**
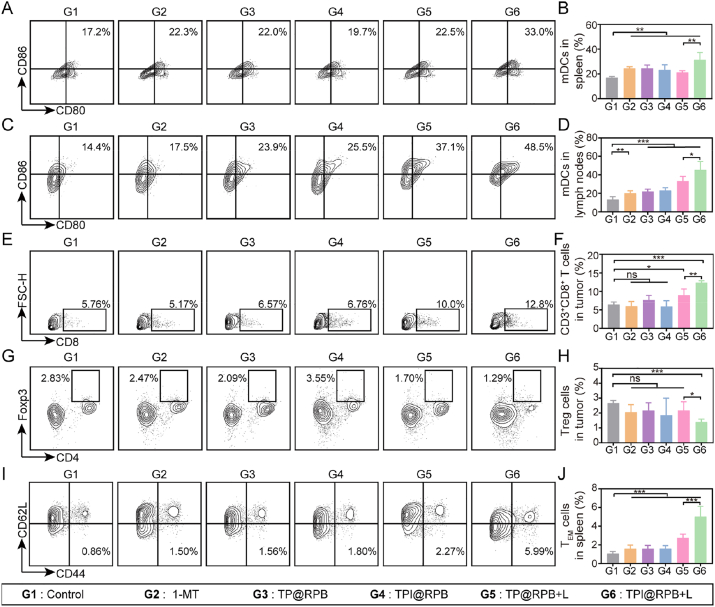
Antitumor immune response *in vivo*. (A) FCM analysis of mature DCs in the spleen in different groups, along with corresponding quantification (B) (N = 5) (representative gating strategy shown in Fig. S16). (C) FCM analysis of mature DCs in lymph nodes in different groups, along with corresponding quantification (D) (N = 5) (representative gating strategy shown in Fig. S16). (E) FCM analysis of CD3^+^CD8^+^ T cells in the tumor in different groups, along with corresponding quantification (F) (N = 5) (representative gating strategy shown in Fig. S17). (G) FCM analysis of Treg cells in the tumor in different groups, along with corresponding quantification (H) (N = 4) (representative gating strategy shown in Fig. S17). (I) FCM analysis of T_EM_ cells in the spleen in different groups, along with corresponding quantification (J) (N = 3) (representative gating strategy shown in Fig. S18). Data were shown as mean ± SD, ns means no statistical significance, ∗ is *p* < 0.05, ∗∗ is *p* < 0.01, ∗∗∗ is *p* < 0.001.

To evaluate the systemic immune response elicited by different treatments, we performed comprehensive FCM analysis of spleens, tumor-draining lymph nodes (TDLNs), and tumor tissues. First, the maturation status of DCs, the major antigen-presenting cells that bridge innate and adaptive responses, was analyzed. As shown in Fig. 8A and B, the number of mature DCs in the spleen was significantly increased in the TPI@RPB + L group and was 1.47-fold higher than in the TP@RPB + L group. This result is consistent with the increase in the percentage of mature DCs observed in the TDLNs after TPI@RPB + L treatment (Fig. 8C and D). Taken together, these results suggest that TPI@RPB + L significantly increased tumor immunogenicity and promoted DC maturation *in vivo*.

FCM analysis showed that treatment with the TPI@RPB + L group resulted in a marked rise in the percentage of CD8^+^ T cells (Fig. 8E and F) in the tumor-infiltrating lymphocyte population relative to the control. Specifically, the CD8^+^ T cell infiltration induced by the TPI@RPB group was not significantly different from that of the control group, but when combined with light, the CD8^+^ T cell infiltration increased 1.92-fold compared with that of the control group; it was also 1.37-fold greater than that of the TP@RPB + L group, indicating a superior capacity to promote cytotoxic T cell recruitment.

The ability of TPI@RPB to remodel the immunosuppressive tumor microenvironment was then examined by measuring the extent of Treg cell infiltration in tumor tissue [50]. Reductions in Treg-cell infiltration were greatest in the TP@RPB + L and TPI@RPB + L groups relative to the other groups (Fig. 8G–H). Among these, the TPI@RPB + L group showed the largest decrease, with Treg infiltration reduced to 51.7% of control. These results indicate that photodynamic therapy combined with immune checkpoint inhibition can effectively alleviate the immunosuppressive tumor microenvironment.

To assess the persistence of anti-tumor immunity, an immune-memory T cell assay was performed to demonstrate the important role of T effector memory cells (T_EM_ cells) in long-term tumor control [51]. As shown in Fig. 8I–J, the percentage of T_EM_ cells in the spleen significantly increased 4.70-fold from 1.07 ± 0.22% in the control group to 5.02 ± 1.11% in the TPI@RPB + L group, indicating that this immunotherapy effectively induced memory T cell generation.

To evaluate whether TPI@RPB + L could induce protective antitumor immune memory, a 4T1 tumor re-challenge model was established (Fig. S19A). During the primary tumor treatment phase, TPI@RPB + L produced the most pronounced tumor growth inhibition, maintaining the average tumor volume at a low level throughout the observation period (Fig. S19B). Individual tumor growth curves further showed complete regression in 3 of 5 mice in the TPI@RPB + L group, compared with 1 of 5 mice in the TP@RPB + L group, whereas no complete response was observed in the non-irradiated groups (Fig. S19D). No obvious body weight loss was detected during treatment, indicating acceptable preliminary tolerability (Fig. S19C). On day 28, mice with complete tumor regression were re-challenged with 4T1 cells. Compared with untreated mice, the TPI@RPB + L group showed marked suppression of re-challenged tumor growth, with a stronger inhibitory effect than the TP@RPB + L group (Fig. S19E). Flow cytometric analysis further revealed a significantly increased proportion of splenic CD8^+^ T_EM_ cells of the TPI@RPB + L group after tumor re-challenge (Fig. S19F–G). These results suggest that TPI@RPB + L not only effectively controls primary tumor growth but may also promote the establishment of antitumor immune memory, thereby enhancing resistance to subsequent tumor re-challenge.

To further examine the local phototoxic response of normal skin to TPI@RPB-mediated laser irradiation, skin tissues from healthy BALB/c mice were collected for histological and immunofluorescence analyses. As shown in Figure S20, H&E staining did not reveal apparent epidermal disruption, tissue necrosis, or obvious inflammatory lesions in the L or TPI@RPB + L groups compared with the control group. TUNEL staining showed no evident increase in apoptotic signals after laser irradiation or TPI@RPB + L treatment. In addition, 4-HNE and Ly6G immunofluorescence staining did not show marked enhancement of lipid peroxidation or neutrophil infiltration in the treated skin tissues. These observations suggest that, under the tested irradiation and dosing conditions, TPI@RPB + L did not induce overt histological photodamage in normal skin.

In addition, the *in vivo* biocompatibility of TPI@RPB was further evaluated. Specifically, major organs (heart, liver, spleen, lungs, and kidneys) were collected from mice 15 days after TPI@RPB injection and examined by H&E staining. Histological examination revealed no evident tissue damage in the major organs after the administration of TPI@RPB, in comparison with the control group (Fig. S21, Supporting Information), indicating good systemic biocompatibility. Additionally, hematological and biochemical parameters were measured in mouse serum, and the TPI@RPB + L groups did not differ significantly from the control group (Fig. S22, Supporting Information), further indicating the favorable *in vivo* biocompatibility and safety profile of TPI@RPB.

Taken together, these findings suggest that co-delivery of an ER-targeted photosensitizer and an IDO-1 inhibitor can effectively reshape the tumor immune microenvironment and enhance antitumor control. Nevertheless, because bilateral tumor and lung metastasis models were not included in the present study, the efficacy of TPI@RPB + L against distant untreated tumors or metastatic lesions requires further validation. At the same time, we recognize that several key considerations remain important as this strategy moves forward. In particular, advancing an RGD-PEG-BSA nanoplatform toward GMP-compliant production will require stringent control over batch-to-batch reproducibility and storage stability. In addition, although 660 nm irradiation represents a practical and widely used trigger, its limited tissue penetration may constrain applications in deep-seated tumors, motivating future efforts to integrate clinically compatible light-delivery strategies. Finally, repeated administration may elicit anti-carrier immune responses, underscoring the need for systematic immunogenicity evaluation in conjunction with longer-term safety studies. Addressing these considerations will help to more clearly define the clinical contexts in which this platform may provide meaningful benefit.

## 3 Conclusion

Herein, this study presents a multifunctional nanoparticle (TPI@RPB) engineered to co-deliver an ER-targeted photosensitizer (TSE-Ppa) and an IDO-1 inhibitor (1-MT) via an RGD-PEG-BSA-based tumor-targeting carrier, aiming to synergistically augment photodynamic immunotherapy for TNBC. TSE-Ppa showed ER-associated localization and, upon 660 nm irradiation, promoted intracellular ROS generation, leading to pronounced ER stress and ICD. Compared with the TPI@RPB group, treatment with the TPI@RPB + L group led to a 4.4-fold increase in extracellular ATP release, along with significantly enhanced CRT exposure and HMGB1 translocation. In parallel, Western blotting and immunofluorescence analysis confirmed that TPI@RPB efficiently downregulated IDO-1 expression, while HPLC quantification revealed a reduction in Kyn production, collectively contributing to the reversal of tumor-induced immunosuppression and supporting ICD-mediated immune activation. In 4T1 tumor-bearing mice, our work demonstrated that the TPI@RPB + L group reduced tumor volume and weight to 16.07% and 13.70% of those in the control group, respectively. Immune phenotype analysis showed that, compared with the control group, the TPI@RPB + L group promoted dendritic-cell maturation and increased CD8^+^ T-cell infiltration to 1.92-fold of the control. Meanwhile, the number of effector memory T cells increased, and regulatory T-cell infiltration decreased to 51.7% of the control. Overall, these results support a synergistic strategy: combining endoplasmic-reticulum–localized oxidative stress with metabolic immune checkpoint blockade to enhance the anti-tumor immune response. The nanoparticle shows clear translational potential for precise immunophotodynamic therapy of triple-negative breast cancer and may also apply to other immunosuppressive solid tumors.

## CRediT authorship contribution statement

**Jia Wang:** Conceptualization, Data curation, Formal analysis, Investigation, Visualization, Writing – original draft. **Fengling Wang:** Conceptualization, Investigation, Methodology, Writing – review & editing. **Qiang Zhou:** Data curation, Formal analysis, Visualization. **Yue Huang:** Formal analysis, Investigation. **Mengjun Yu:** Investigation, Methodology. **Shiwen Chen:** Investigation, Methodology. **Jing Gong:** Investigation, Methodology. **Min Yang:** Investigation, Methodology. **Qiang He:** Investigation, Methodology. **Jingbin Huang:** Conceptualization, Funding acquisition, Writing – review & editing. **Yu Zhao:** Conceptualization, Funding acquisition, Supervision.

## Declaration of competing interest

The authors declare that they have no known competing financial interests or personal relationships that could have appeared to influence the work reported in this paper.

## Data Availability

Data will be made available on request.
