# Supplementary material for: Co-delivery of endoplasmic reticulum-targeted photosensitizer and Ido-1 inhibitor potentiates immunotherapeutic efficacy in triple-negative breast cancer
Source: Mater Today Bio. 2026 May 22;38:103268. doi: 10.1016/j.mtbio.2026.103268 (PMC13241888; doi:10.1016/j.mtbio.2026.103268)
Supplement: Multimedia component 1 [file mmc1.docx]

Supporting Information

**Co-Delivery of Endoplasmic Reticulum-Targeted Photosensitizer and IDO-1 Inhibitor Potentiates Immunotherapeutic Efficacy in Triple-Negative Breast Cancer**

Jia Wang^1, #^, Fengling Wang^2, #^, Qiang Zhou^2^.Yue Huang^2^, Mengjun Yu^2^, Shiwen Chen^1^, Jing Gong^2^, Min Yang^2^, Qiang He^3^, Jingbin Huang^2,^ *, Yu Zhao^1,^ *

1. Department of Pharmacy, University-Town Hospital of Chongqing Medical University, Chongqing 401331, China

2. Department of Pharmacy, The Second Affiliated Hospital of Army Medical University, Chongqing 400037, China

3. Department of Pharmacy, Bishan Hospital of Chongqing Medical University, Chongqing 402760, China

* Correspondence: sige@tmmu.edu.cn (J.H.); zhaoyuu@hotmail.com (Y.Z.)

\# These authors contributed equally to this work.

**1. Materials and methods**

***1.1 Materials***

N-tosylethylenediamine (TSE, 693855) was purchased from Sigma-Aldrich (U. S.); Pyropheophorbide-a (Ppa, H124006-500 mg) was purchased from D&B (China); RGDfC peptide was purchased from GL Biochem (China) Ltd; Methoxy PEG Succinimidyl Carboxymethyl (mPEG5000-NHS) and MAL-PEG5000-NHS were purchased from JenKem (China); 1-hydroxybenzotriazole (HOBt), Sodium chloride (NaCl, AR), Dichloromethane (DCM, AR), DMSO (AR), Petroleum ether (PE, AR) , Acetone (AR) and Glacial acetic acid (AR) were purchased from Chuan Dong Chemical Group (China); Trifluoroacetic acid (TFA, AR) was purchased from Shanghai Acmec Biochemical Technology Co. , Ltd (China); Acetonitrile (HPLC) was purchased from Honeywell (U. S. ); 1-ethyl-3-(3-dimethylaminopropyl) carbodiimide (EDC, A638729), Triethylamine (TEA, T103285), 1-methyl-tryptophan (1-MT, M138479) and 1,3-diphenylisobenzofuran (DPBF, D122454) were purchased from Aladdin (China); IFN-γ(MA0620), Sodium acetate (MB2529-1) and L-kynurenine hydrate (HPLC, MB5637-1) were purchased from Dalian Meilun Biotechnology Co. , Ltd (China); Bovine serum albumin (BSA, ST2254), 4',6-diamidino-2-phenylindole (DAPI, C1005), 4% polyformaldehyde fixing solution (P0099), ROS assay kit (S0033S), Cell counting kit-8 (CCK-8, MA0218-500T), Calcein-AM/PI cell live/dead assay kit (C2015M), ATP assay kit (S0026), ACK lysis buffer (C3702) and One step TUNEL apoptosis assay kit (C1090) were purchased from Beyotime (China); PDI Polyclonal antibody (11245-1-AP), Phospho-PERK/EIF2AK3 (Thr982) Recombinant monoclonal antibody (82534-1-RR) and GRP78/BIP Polyclonal antibody (11587-1-AP) were purchased from Proteintech (China); LAMP1 Recombinant Rabbit Monoclonal Antibody [PSH05-55] (HA722302) and DDIT3 Recombinant Rabbit Monoclonal Antibody [PSH07-65] (HA722854) were purchased from HUABIO (China);Alexa Fluor®488 Rabbit monoclonal [EPR3507] to HMGB1 (AB195010) and Goat Anti-Rabbit IgG H&L (Alexa Fluor® 488) (ab150077) were purchased from Abcam (U. K. ); Mouse High Mobility Group Protein 1 (HMGB1) ELISA Kit (JL13702) was purchased from JONLNBIO (China); Calreticulin (D3E6) XP® rabbit mAb (12238S) and IDO (D8W5E) rabbit mAb (51851) were purchased from Cell Signaling Technology, Inc (U. S. ); IDO-1 recombinant rabbit monoclonal antibody (AWA10272-20ul) was purchased from Changsha Abiowell Biotechnology Co. , Ltd (China); Zombie Red^TM^ Fixable Viability Kit (423110), APC/Fire^TM^ 750 anti-mouse CD45 (30-F11), PE/Cy7 anti-mouse CD11c (N418), PerCP/Cyanine5.5 anti-mouse I-A/I-E Antibody (107626), APC anti-mouse CD80 (104714), BV421 anti-mouse CD86 (105032), BV510 anti-mouse CD3 (100234), FITC anti-mouse CD8a (100706), APC anti-mouse CD44 (103012), PE/Cyanine 7 anti-mouse CD62L (MEL-14), BV650 anti-mouse CD4 (100545) and Brilliant Violet 421^TM^ anti-mouse Foxp3 (MF-14) were purchased from Biolegend (U. S.). The mouse triple-negative breast cancer cell line (4T1) was obtained from iCell (China) and was cultivated in 1640 medium containing 10% fetal bovine serum (ST11-001S, Lonsera, China) and 1% pen-strep (S110JV, BasalMedia, China) at 37 ℃ with 5% CO_2_. Eight-week-old female BALB/c mice and C57 mice were obtained from Hunan SJA Laboratory Animal Co.， Ltd (China). All animal procedures were carried out under the guidelines approved by the Laboratory Animal Welfare and Ethics Committee of the Army Medical University (AMUWEC20245297).

***1.2 Synthesis of TSE-Ppa and preparation of TPI@RPB***

*1.2.1 Synthesis of TSE-Ppa*

A total of 120 mg of TSE and 200 mg of Ppa were dissolved in 100 mL of DCM in a 250 mL round-bottom flask. Catalysts, including 144 mg of EDC, 101 mg of HOBt, and 30 μL of TEA, were added. The reaction was stirred for 24 h and then washed with 100 mL of deionized water containing NaCl. The crude product was purified via silica gel column chromatography using petroleum ether: acetone (2:1) as the eluent, followed by rotary evaporation to obtain TSE-Ppa. The final product was characterized by using ^1^H-NMR (Quantum-I Plus, Q.One Instruments Ltd., China) and Matrix-Assisted Laser Desorption/Ionization Time-of-Flight Mass Spectrometry (MALDI-TOF MS, autoflex speed, Bruker, Germany).

*1.2.2 Preparation of RGD-PEG-BSA and mPEG-BSA*

RGDfC (17 mg) was reacted with MAL-PEG5000-NHS (147 mg) in PBS, followed by the addition of BSA (400 mg) with continued stirring at room temperature for 12 h. The reaction mixture was dialyzed and lyophilized to obtain the targeted polymer RGD-PEG-BSA. As a control, mPEG-BSA was obtained by reacting BSA (400 mg) with mPEG5000-NHS (150 mg) in PBS at room temperature for 2 h, followed by dialysis and freeze-drying. The final products were characterized by using ^1^H-NMR (Quantum-I Plus, Q.One Instruments Ltd., China), Fourier Transform Infrared Spectroscopy (FTIR, IRTracer-100, SHIMADZU, Japan), and MALDI-TOF MS.

*1.2.3 Preparation of TPI@RPB*

1-MT and TSE-Ppa were respectively dissolved in DMSO, and RGD-PEG-BSA was dissolved in ultrapure water to obtain stock solutions with a concentration of 10 mg/mL. A mixture of 40 μL 1-MT, 40 μL TSE-Ppa was prepared, followed by dilution of 200 μL RGD-PEG-BSA to 5 mL with ultrapure water. The solution was then subjected to ice bath ultrasonication (20 kHz, 3 min, 3 s on/3 s off) while simultaneously adding the mixture of 1-MT and TSE-Ppa dropwise. The resulting TPI@RPB was obtained post-ultrasonication.

The preparation of TP@RPB followed an analogous procedure, with the modification that 40 μL TSE-Ppa was loaded individually in the absence of 1-MT.

TPI@PB was prepared using the same procedure as TPI@RPB, except that the non-c(RGDfC)-modified control carrier mPEG-BSA was used instead of RGD-PEG-BSA to encapsulate identical amounts of TSE-Ppa and 1-MT.

PI@RPB was prepared by co-loading Ppa, at a molar amount equivalent to that of TSE-Ppa, and the same amount of 1-MT into the RPB carrier under the same preparation conditions as TPI@RPB.

TPI@PB/IR780 and TPI@RPB/IR780 were prepared by additionally encapsulating 10 μL of IR780 solution (10 mg/mL) into TPI@PB and TPI@RPB, respectively.

***1.3 Characterization of nanoparticles***

The particle size, polydispersity index (PDI), and zeta potential of nanoparticles were characterized by dynamic light scattering (DLS, Malvern Zetasizer Nano ZS90, U.K.), while their morphology was examined using transmission electron microscopy (TEM, JEM-1400Plus, JEOL, Japan). Before characterization, the nanoparticles were dialyzed for 12 h. The encapsulation efficiency (EE%) and drug loading efficiency (LE%) were quantified via UV-Vis spectroscopy (Evolution 201, ThermoFisher, U.S.) and high-performance liquid chromatography (HPLC, Agilent 1200, Santa Clara, California, U.S.) using the following equations:

EE (%) = (mT − mU)/mT × 100%
LE (%) = (mT − mU)/mZ × 100%

where mT is the total weight of TSE-Ppa (or 1-MT), mU is the weight of unencapsulated drug, and mZ is the total weight of nanoparticles. Nanoparticles were stored at room temperature, and their particle size, PDI, and zeta potential were monitored at 1, 2, 3, 4, 5, 6, and 7 days.

TPI@RPB and TP@RPB nanoparticles were prepared as described above. After ultrafiltration, the nanoparticles were resuspended in PBS or RPMI 1640 medium supplemented with 10% fetal bovine serum, followed by incubation in a thermostatic shaker at 37 °C and 120 rpm. Changes in particle size, polydispersity index, and zeta potential were monitored at 0, 3, 6, 9, 12, and 24 h.

The generation of ROS was assessed by using the DPBF probe, as the ROS could reduce the absorption of DPBF. A 50 μL DPBF solution (1 mM) was mixed with 3 mL of TPI@RPB suspension (100 μg/mL). The absorbance spectra (300–500 nm) of the laser-treated mixture (660 nm, 0.5 W/cm²) were recorded before irradiation and every 20 seconds for 2 min using UV-Vis spectroscopy. Two groups were established for comparison: DPBF+Laser, TPI@RPB+DPBF.

***1.4* *Hemocompatibility studies***

Whole blood from healthy C57 mice was collected, and red blood cells (RBCs) were isolated by centrifugation (3000 rpm, 5 min). The RBCs were washed with PBS until no visible red color remained in the supernatant and then diluted to 10%. RBC suspensions (0.3 mL) were mixed with RGD-PEG-BSA solutions at different concentrations (10–640 μg/mL). Negative control (RBCs in PBS) and positive control (RBCs in deionized water) were included. The samples were shaken in a thermostatic incubator at 37 ℃ for 3 h, centrifuged, and the supernatants were photographed before being analyzed for absorbance at 414 nm to calculate the hemolysis rate (Multiskan SkyHigh, ThermoFisher, U.S.).

***1.5 In vitro 1-MT release***

The in vitro release behavior of 1-MT from TPI@RPB was evaluated using a dialysis method under different pH and irradiation conditions. Briefly, TPI@RPB dispersion was sealed in dialysis bags with an appropriate molecular weight cut-off and immersed in PBS release media at pH 7.4, pH 6.5, or pH 5.0. According to the experimental design, five release conditions were established: pH 7.4, pH 6.5, pH 6.5+L, pH 5.0, and pH 5.0+L. For the light-treated groups, TPI@RPB was exposed to light irradiation under the preset irradiation conditions before being subjected to the corresponding release medium.

All samples were incubated in a thermostatic shaker at 37 °C with gentle agitation. At predetermined time points of 0.5, 1, 2, 4, 6, 8, 12, 24, and 48 h, an aliquot of the external release medium was collected and replaced with an equal volume of fresh medium with the same pH. The concentration of released 1-MT was quantified by HPLC using a standard calibration curve of 1-MT. The cumulative release percentage of 1-MT was calculated according to the following equation:

$$\text{Cumulative release(\%)=}\frac{\text{V}_{\text{0}}\text{C}_{\text{n}}\text{+}\text{V}_{\text{s}}\text{∑}_{\text{i=1}}^{\text{n-1}}\text{C}_{\text{i}}}{\text{W}_{\text{0}}}\text{×}\text{100\%}$$

where V_0_ is the total volume of release medium, V_s_ is the sampling volume, C_n_ is the drug concentration measured at the nth sampling time, C_i_ is the drug concentration measured at the previous sampling points, and W_0_ is the initial amount of drug loaded in the nanoparticles.

***1.6 Cellular uptake and ER localization***

4T1 and L929 cells were seeded into 12-well culture plates at a density of 2 ×105 cells/well and incubated for 24 h, then treated with TPI@RPB or TPI@PB for 0.5,1 ,2, or 4 h, washed with PBS, and analyzed by flow cytometry (FCM, BD LSRFortessa™, Biosciences, U.S.) for fluorescence intensity. For confocal laser scanning microscopy (CLSM, LSM880, Zeiss, Germany), 4T1 cells were then fixed, stained with DAPI, and visualized to assess intracellular distribution.

4T1 cells were seeded into 12-well culture plates at a density of 2 × 105 cells/well and incubated for 24 h. To assess RGD-mediated uptake, cells in the competition groups were preincubated with free c(RGDfC) peptide (200 μM) at 37 °C for 30 min, followed by treatment with TPI@RPB or TPI@PB for 2 h. Cells incubated with TPI@RPB or TPI@PB in the absence of free c(RGDfC) served as non-competitive controls. After treatment, the cells were rinsed with PBS, and analyzed by FCM.

To investigate the internalization pathway of TPI@RPB in 4T1 cells, cells were seeded in 12-well plates and cultured for 24 h. The cells were then pretreated with chlorpromazine (CPZ, 10 μg/mL), nystatin (25 μg/mL), or amiloride (50 μM) at 37 °C for 30 min. A low-temperature group incubated at 4 °C was included as an energy-dependent uptake control. Subsequently, TPI@RPB was added and incubated for another 2 h. After washing with PBS, the cells were analyzed by FCM.

4T1 cells were seeded into 12-well culture plates at a density of 1.5 × 105 cells/well and incubated for 24 h. After treatment with TPI@RPB or PI@RPB for 4 h, the cells were washed with PBS, fixed with 4% paraformaldehyde, permeabilized with 0.1% Triton X-100, and blocked with 5% BSA. The ER was labeled with an anti-PDI primary antibody, and lysosomes were labeled with an anti-LAMP1 primary antibody, followed by incubation with an Alexa Fluor 488-conjugated secondary antibody. Finally, the cells were stained with DAPI and examined by CLSM.

Western blotting was performed to evaluate the expression of ER stress-related proteins in 4T1 cells. Briefly, 4T1 cells were seeded in culture dishes and allowed to adhere for 24 h. The cells were then treated with Control, L, PI@RPB, TPI@RPB, PI@RPB+L, or TPI@RPB+L for 4 h, with the TSE-Ppa concentration fixed at 2.5 μg/mL. For irradiation groups, cells were exposed to a 660 nm laser at 0.5 W/cm² for 5 min. After an additional 4 h incubation, cells were lysed on ice for 30 min using RIPA lysis buffer supplemented with protease and phosphatase inhibitors. The lysates were centrifuged at 13,000 g for 10 min at 4 °C, and the supernatants were collected for protein quantification using a BCA assay. Equal amounts of protein, 30 μg per sample, were separated by 10% SDS-PAGE at 120 V for 90 min and then transferred onto PVDF membranes at 250 mA for 130 min. After blocking with a rapid blocking buffer for 10 min at room temperature, the membranes were washed three times with TBST and incubated with primary antibodies overnight at 4 °C. The membranes were subsequently washed with TBST and incubated with HRP-conjugated anti-mouse or anti-rabbit secondary antibodies for 2 h at room temperature. Protein bands were visualized using an enhanced chemiluminescence (ECL) chemiluminescence detection system.

***1.7 ROS generation***

The ROS generation was assessed using DCFH-DA staining. 4T1 cells were seeded into 12-well culture plates at a density of 1.5 × 10^5^ cells/well and incubated for 24 h. To elucidate the ROS generation of TPI@RPB, 4T1 cells were incubated with 1-MT, TP@RPB, TPI@RPB, TP@RPB+L, and TPI@RPB+L at a TSE-Ppa concentration of 1.25 μg/mL for 4 h, and then the laser treatment groups were irradiated with a 660 nm laser (0.5 W/cm^2^, 3 min). After incubation for 1 h, the cells were washed with PBS, stained with DCFH-DA at 37 ℃ for 20 min, and analyzed by an inverted fluorescence microscope (IFM, DMi8, Leica, Germany). Additionally, FCM was conducted to investigate the ROS generation of TPI@RPB quantitatively.

***1.8 Cytotoxicity***

Cytotoxicity was evaluated using a CCK-8 assay and live/dead staining in 4T1 cells. 4T1 cells were seeded in the 96-well plate at a density of 3500 cells/well in 100 μL of medium and incubated for 24 h. To elucidate the cytotoxicity of TPI@RPB, 4T1 cells were incubated with different concentrations of 1-MT, TP@RPB, TPI@RPB, TP@RPB+L, and TPI@RPB+L for 4 h, and then the laser treatment groups were irradiated with a 660 nm laser (0.5 W/cm^2^, 2 min). After 24 hours, 10 μL CCK-8 was added to each well and incubated at 37 ℃ for 1 h, and the absorbance was measured at 450 nm using a microplate reader to calculate the cell viability. Live/dead staining with calcein-AM and PI was used for further assessment. 4T1 cells were seeded into 12-well culture plates at a density of 1.5 × 10^5^ cells/well and incubated for 24 h. Cells were treated with different treatment agents at a TSE-Ppa concentration of 1.25 μg/mL and incubated for 4 h, and then the laser treatment groups were irradiated with a 660 nm laser (0.5 W/cm^2^, 3 min). After 24 hours, the cells were washed with PBS, stained with calcein-AM and PI at 37 ℃ for 30 min, and analyzed by IFM.

***1.9 Enhancement of ICD***

*1.9.1 CRT exposure assay*

The surface exposure of CRT was assessed via immunofluorescence staining. 4T1 cells were seeded into 12-well culture plates at a density of 1.5 × 10^5^ cells/well and incubated for 24 h. The cells were then treated with different treatment agents (TSE-Ppa concentration: 2.5 μg/mL) for 4 h, and then the laser treatment groups were irradiated with a 660 nm laser (0.5 W/cm^2^, 3 min). After an additional 4 h of incubation, the cells were fixed with 4% paraformaldehyde for 15 min. Next, the cells were blocked with 1% BSA in PBS for 30 min, and incubated with the primary antibody overnight at 4℃. The cells were then incubated with the Alexa488-conjugated monoclonal secondary antibody for 2 h. Finally, the cells were stained with DAPI and examined by CLSM.

*1.9.2 Intracellular HMGB1 translocation assay*

The intracellular distribution of HMGB1 was evaluated using immunofluorescence under conditions identical to the CRT assay, with slight modifications. Following fixation (4% paraformaldehyde, 15 min), the cells were permeabilized with 0.1% Triton X-100 for 10 min, and blocked with 1% BSA in PBS for 30 min. Subsequently, the cells were incubated with HMGB1 antibody overnight at 4℃, stained with DAPI, and examined by digital pathology slide scanner (KF-FL-020, KFBIO, China). In parallel, HMGB1 released into the culture medium was quantified by ELISA. Briefly, culture supernatants were collected from each group, centrifuged to remove residual cells and debris, and subjected to HMGB1 ELISA following the manufacturer’s protocol.

*1.9.3 Extracellular ATP secretion assay*

Extracellular secretion of ATP was tested using a commercially available ATP assay kit. 4T1 cells were seeded into 12-well culture plates at a density of 2 × 10^5^ cells/well and incubated for 24 h. After treatment with different treatment agents (TSE-Ppa concentration: 2.5 μg/mL, 4 h) and then the laser treatment groups were irradiated with a 660 nm laser (0.5 W/cm^2^, 3 min). The cell culture supernatant was collected 6 h post-irradiation. ATP levels were tested using an ATP assay kit according to the manufacturer’s instructions.

*1.9.4 DCs' maturation assay*

Bone marrow-derived dendritic cells (BMDCs) were obtained from the femurs and tibias of C57BL/6 mice and cultured in RPMI-1640 medium supplemented with 10% fetal bovine serum, 10 ng/mL GM-CSF, and 5 ng/mL IL-4. Half of the culture medium was refreshed every two days, and immature DCs were collected on day 6 for subsequent experiments.4T1 cells were seeded in transwell inserts, pretreated with different agents (TSE-Ppa, 2.5 μg/mL, 4h), and then the laser treatment groups were irradiated with a 660 nm laser (0.5 W/cm^2^, 3 min) before co-culture with BMDCs for 24 hours. Cells were then stained with APC/Cy7 anti-mouse CD45, PE/Cy7 anti-mouse CD11c, FITC anti-mouse CD80, and APC anti-mouse CD86 antibodies, and analyzed for maturation by FCM.

***1.10 Evaluation of IDO-1 inhibition by TPI@RPB nanoparticles***

*1.10.1 Immunofluorescence analysis of intracellular IDO-1 distribution*

Immunofluorescence staining was performed to examine the intracellular localization of IDO-1. 4T1 cells were seeded into 12-well culture plates at a density of 1.5 × 10^5^ cells/well and incubated for 24 h. All groups were stimulated with IFN-γ (100 ng/mL) for 24 h before treatment. Then the cells were treated with 1-MT, TP@RPB, TPI@RPB (1-MT concentration:10 μg/mL) for 24 h. Following fixation (4% paraformaldehyde, 15 min). The cells were blocked with 1% BSA in PBS for 30 min, and incubated with the primary antibody overnight at 4℃. The cells were then incubated with the Alexa488-conjugated monoclonal secondary antibody for 2 h. Finally, the cells were stained with DAPI and examined by CLSM.

*1.10.2 Western Blot analysis of IDO-1 expression*

The intracellular expression of IDO-1 was assessed via Western Blot Analysis. 4T1 cells were seeded in culture dishes and incubated for 24 h. All groups were stimulated with IFN-γ (100 ng/mL) for 24 h before treatment. The cells were treated with 1-MT, TP@RPB, TPI@RPB, TP@RPB+L, and TPI@RPB+L (1-MT concentration:10 μg/mL) for 4 h, and then the laser treatment groups were irradiated with a 660 nm laser (0.5 W/cm^2^, 5 min). After 24 h incubation, Cells were lysed in 50 μL RIPA buffer supplemented with protease inhibitors on ice for 30 min. Lysates were centrifuged at 13000 g for 10 min at 4°C, and supernatants were collected for protein quantification using a BCA assay. Proteins (20 μg per lane) were separated by 10% SDS-PAGE gels at 120 V for 90 min under constant voltage and transferred to PVDF membranes (250 mA, 70 min). Membranes were blocked with Rapid Block Buffer for 10 min at room temperature, washed three times with TBST, and incubated with primary antibodies overnight at 4°C. After three TBST washes, membranes were incubated with HRP-conjugated anti-mouse/anti-rabbit secondary antibodies for 2 h at room temperature. Finally, protein bands were visualized using an ECL detection system.

*1.10.3 HPLC quantification of Kyn production*

4T1 cells were evenly seeded in a 24-well plate and incubated for 24 h. All groups were stimulated with IFN-γ (100 ng/mL) for 24 h before treatment. The cells were treated with 1-MT, TP@RPB, TPI@RPB (1-MT concentration:10 μg/mL) for 4 h, and then the laser treatment groups were irradiated with a 660 nm laser (0.5 W/cm^2^, 3 min). After 24 h incubation, 140 μL of the supernatant was mixed with 10 μL of trifluoroacetic acid (TCA, W/V) and incubated in a constant-temperature oven at 50°C for 30 min. The mixture was then centrifuged at 10000 rpm for 10 min to remove the precipitate. The supernatant was collected from each EP tube for HPLC analysis. (HPLC conditions: column: Phenomenex Luna 5u C18 100A (250 mm × 4.6 mm, 5 μm); mobile phase: acetonitrile/sodium acetate solution (8:92, v/v); flow rate: 1.0 mL/min; detection wavelength for Kyn: 360 nm; column temperature: 30°C; injection volume: 20 μL).

***1.11* *In Vivo evaluation of nanoparticles***

*1.11.1 Tumor targeting via in vivo imaging*

To evaluate tumor-targeting capability, IR780-labeled (Ex: 777, Em: 823) TPI@RPB and TPI@PB were administered intravenously to 4T1 tumor-bearing mice. Whole-body fluorescence imaging was performed at 3, 6, 12, 24, 36, and 48 h post-injection (IVIS Lumina LT, Perkin Elmer, U.S.). *Ex vivo* imaging of organs and tumors was conducted at 48 h.

*1.11.2 Antitumor efficacy assessment*

Female BALB/c mice were subcutaneously inoculated with 4T1 cells on day −4 to establish the primary tumor model. When the tumors reached the appropriate volume (mean tumor volume ≈ 50 mm³, calculated as length × width² × 0.5), thirty mice were randomized into six groups (N = 5): Control, 1-MT, TP@RPB, TPI@RPB, TP@RPB+L and TPI@RPB+L. The formulations were intravenously administered at an equivalent TSE-Ppa dose of 5 mg/kg on days 0, 2, and 4. For the irradiation groups, tumors were exposed to a 660 nm laser (0.5 W/cm², 5 min) on days 1, 3, and 5 after injection. Tumor dimensions were measured every other day using digital calipers, and body weights were recorded concurrently using an analytical balance.

*1.11.3 Immunological mechanism evaluation*

After the final treatments, the tumors, spleens, and lymph nodes were harvested and stained with an antibody to investigate the *in vivo* immune response elicited by TPI@RPB.

Tumor tissue sections were first subjected to hematoxylin and eosin (H&E) staining for histopathological evaluation. Adjacent sections were then sequentially subjected to multiplex fluorescence staining for terminal deoxynucleotidyl transferase dUTP nick-end labeling (TUNEL), CRT, HMGB1, and IDO-1, and examined by CLSM and IFM.

Spleens, tumors, and draining lymph nodes were collected from mice. Tissues were mechanically dissociated to prepare single-cell suspensions, which were passed through 70-μm cell strainers to remove debris. Red blood cells were lysed using ACK lysis buffer. All samples were first stained with Zombie Red™ Fixable Viability Kit and APC/Fire™750 anti-mouse CD45 to exclude dead cells and identify leukocytes. For the analysis of mature DCs (CD11c⁺MHC II⁺CD80⁺CD86⁺), single-cell suspensions from spleens and lymph nodes were further stained with PE/Cy7 anti-mouse CD11c, PerCP/Cyanine5.5 anti-mouse I-A/I-E, APC anti-mouse CD80, and BV421 anti-mouse CD86. For the analysis of CTLs (CD3⁺ CD8a⁺), tumor-derived cells were stained with BV510 anti-mouse CD3, and FITC anti-mouse CD8a. For the analysis of Treg cells (CD3⁺CD4⁺Foxp3⁺), tumor-derived cells were stained with BV510 anti-mouse CD3, BV650 anti-mouse CD4, and BV421 anti-mouse Foxp3. For the analysis of effector memory T cells (T_EM_; CD3⁺CD8a⁺CD44⁺CD62L⁻), spleen-derived cells were stained with BV510 anti-mouse CD3, FITC anti-mouse CD8a, APC anti-mouse CD44, and PE/Cy7 anti-mouse CD62L. All stained cells were analyzed using FCM.

*1.11.4 Tumor re-challenge experiment*

The primary 4T1 tumor model and treatment schedule were established as described in the in vivo antitumor study. Mice showing complete regression or marked control of the primary tumor after the initial treatment were selected for the subsequent tumor re-challenge assay. On day 28, the cured mice were subcutaneously re-inoculated with 4T1 cells at a distant site without additional treatment. Age-matched untreated mice receiving the same 4T1 inoculation served as controls. Tumor growth at the re-challenge site was monitored until day 42. At the endpoint, spleens were harvested for flow cytometric analysis of CD8⁺ effector memory T cells.

*1.11.5 In vivo safety assessment*

To evaluate the potential phototoxicity of nanoparticles toward normal skin under laser irradiation, healthy tumor-free female BALB/c mice were used. The skin over the right second mammary fat pad region was shaved and depilated. Mice were randomly divided into three groups: untreated Control, laser irradiation alone (L), and TPI@RPB combined with laser irradiation (TPI@RPB+L). Mice in the TPI@RPB+L group received TPI@RPB via tail vein injection at the same dose as described in Antitumor efficacy assessment. At 24 h post-injection, the depilated skin regions of mice in the L and TPI@RPB+L groups were exposed to a 660 nm laser at 0.5 W/cm² for 5 min. At 24 h after irradiation, mice were euthanized, and skin tissues from the irradiated area were collected. The samples were fixed in 4% paraformaldehyde, embedded in paraffin, and sectioned for subsequent histological and immunofluorescence analyses. H&E staining was performed to examine tissue morphology, fluorescent TUNEL staining was used to detect apoptotic cells, 4-hydroxynonenal (4-HNE) immunofluorescence staining was conducted to assess lipid peroxidation, and Ly6G immunofluorescence staining was applied to evaluate neutrophil infiltration.

In addition, at the end of the treatment regimen described in Section *1.11.2*, blood samples were collected from mice in each group for hematological and biochemical analysis. Subsequently, major organs, including the heart, liver, spleen, lungs, and kidneys, were harvested and subjected to histopathological examination by H&E staining.

***1.12* *Statistical analysis***

All experiments were performed independently at least 3 times. Statistical analyses were performed using GraphPad Prism 8.0.2. (GraphPad Software, Inc.). The difference between the two groups was analyzed by a two-tailed Student's t-test. For differences between multiple groups, a one-way analysis of variance was used. The data was expressed as mean standard deviation. Ns means no statistical significance, * is *p* < 0.05, ** is *p* < 0.01, *** is *p* < 0.001.

**Figure S1.** The synthetic route of TSE-Ppa.

**Figure S2.** The ^1^H-NMR spectrum of TSE-Ppa. ^1^H NMR (600 MHz, DMSO-d6) δ 9.65 (s, 1H), 8.88 (s, 1H), 8.19 (dd, J = 17.8, 11.6 Hz, 1H), 7.84 (t, J = 5.9 Hz, 1H), 7.59 (d, J = 7.9 Hz, 2H), 7.51 (t, J = 6.1 Hz, 1H), 7.27 (d, J = 7.9 Hz, 2H), 6.37 (d, J = 17.8 Hz, 1H), 6.19 (d, J = 11.6 Hz, 1H), 5.21 (d, J = 19.5 Hz, 1H), 5.08 (d, J = 19.4 Hz, 1H), 4.54 (d, J = 9.0 Hz, 2H), 4.26 (d, J = 9.1 Hz, 1H), 3.65 (q, J = 7.8 Hz, 2H), 3.59 (s, 3H), 3.17 (s, 3H), 3.05 (dp, J = 19.7, 6.7 Hz, 2H), 2.70 (q, J = 6.6 Hz, 2H), 2.48 (s, 1H), 2.22 (s, 3H), 2.07 (dd, J = 19.0, 6.9 Hz, 1H), 1.78 (d, J = 7.3 Hz, 3H), 1.60 (t, J = 7.7 Hz, 3H).

**Figure S3.** The MALDI-TOF MS of TSE-Ppa.

**Figure S4.** The synthetic route of RGD-PEG-BSA.

**Figure S5.** The ^1^H-NMR spectrum of C(RGDfC), MAL-PEG5000-NHS, BSA, and RGD-PEG-BSA.

**Figure S6.** The FTIR spectrum of C(RGDfC), MAL-PEG5000-NHS, BSA, and RGD-PEG-BSA.

B

A

**Figure S7.** (A) The MALDI-TOF MS of RGD-PEG-NHS. (B) The MALDI-TOF MS of BSA and RGD-PEG-BSA.

A

B

**Figure S8.** (A) The UV spectra of TSE-Ppa and 1-MT. (B) HPLC chromatographic profile of 1-MT.

**Figure S9.** Changes in hydrodynamic diameter (A), PDI (B), and zeta-potential (C) of TPI@RPB and TP@RPB after one week of storage in deionized water.

B

A

**Figure S10.** (A) Time-dependent absorbance changes of DPBF at 300 - 500 nm under laser irradiation (660 nm, 0.5 W/cm2) in the DPBF+L group. (B) Time-dependent absorbance changes of DPBF at 300 - 500 nm in the TPI@RPB+DPBF group.

**Figure S11.**Cumulative release profiles of 1-MT from TPI@RPB at pH 7.4, pH 6.5, and pH 5.0 with or without laser irradiation (N = 3).

B

**Figure S12.** (A) Intracellular uptake of TPI@RPB and TPI@PB by 4T1 cells with CLSM at different durations, scale bar = 20 µm. (B) Quantification of intracellular mean fluorescence intensity in panel A (N = 3).

**Figure S13.** Flow cytometric analysis of TPI@RPB uptake in 4T1 cells after pretreatment with different endocytosis inhibitors or incubation at 4 °C (N = 3).

**Figure S14.** HMGB1 release from 4T1 cells upon each treatment was quantified by ELISA (N = 3).

**Figure S15.** Tumor growth curves in the Control group (A), 1-MT group (B), TP@RPB group (C), TPI@RPB group (D), TP@RPB+L group (E), and TPI@RPB+L group (F) of mice (N = 5).

**Figure S16.** Single-cell suspensions were first gated to exclude debris and doublets based on FSC/SSC parameters. Live CD45⁺ leukocytes were then selected, followed by gating of CD11c⁺MHC-II⁺ dendritic cells. Mature DCs were identified as CD80⁺CD86⁺ cells within the CD11c⁺MHC-II⁺ population.

**Figure S17.** Single-cell suspensions were first gated to exclude debris and doublets based on FSC/SSC parameters. Live CD45⁺ leukocytes were then selected, followed by gating of CD3⁺ T cells. CD8⁺ T cells were identified as CD3⁺CD8⁺ cells, and regulatory T cells were defined as CD3⁺CD4⁺Foxp3⁺ cells.

**Figure S18.** Single-cell suspensions were first gated to exclude debris and doublets based on FSC/SSC parameters. Live CD45⁺ leukocytes were then selected, followed by gating of CD3⁺CD8⁺ T cells. CD8⁺ effector memory T cells were defined as CD44⁺CD62L⁻ cells within the CD3⁺CD8⁺ population.

**Figure S19.** Tumor re-challenge assay for evaluating long-term antitumor immune memory. (A) Schematic illustration of the tumor re-challenge experimental timeline. (B) Primary tumor volume curves of mice in each treatment group. (C) Body weight changes of mice during treatment. (D) Individual primary tumor growth curves of mice in each treatment group. CR indicates complete tumor regression; values indicate the number of mice with complete tumor regression out of the total number of mice in each group. (E) Tumor growth curves after 4T1 re-challenge. (F–G) Representative flow cytometry plots and quantification of splenic CD8⁺ effector memory T cells (T_EM_, CD3⁺CD8⁺CD44⁺CD62L⁻) after tumor re-challenge (N=3).

**Figure S20.** Evaluation of laser irradiation-associated phototoxicity in normal skin tissues. Representative images of H&E staining (A), TUNEL staining (B), 4-HNE immunofluorescence staining (C), and Ly6G immunofluorescence staining (D). Scale bar = 100 μm.

**Figure S21.** H&E- stained sections of the main organs of mice in different groups. Scale bars = 100 μm.

**Figure S22.** Main hematological and biochemical parameters of mice in different groups (N = 3-5). Data are shown as mean ± SD. **Ns means no statistical significance.**
